# Creation of an Automated and Comprehensive Resident Progress System for Residents and to Save Hours of Faculty Time: Mixed Methods Study

**DOI:** 10.2196/53314

**Published:** 2024-09-23

**Authors:** Rimma Perotte, Alyssa Berns, Lana Shaker, Chayapol Ophaswongse, Joseph Underwood, Christina Hajicharalambous

**Affiliations:** 1 Hackensack University Medical Center Hackensack, NJ United States; 2 Hackensack Meridian School of Medicine Nutley, NJ United States; 3 Columbia University Medical Center New York, NY United States

**Keywords:** progress dashboard, informatics in medical education, residency learning management system, residency progress system, residency education system, summarization, administrative burden, medical education, resident, residency, resident data, longitudinal, pilot study, competency, dashboards, dashboard, faculty, residents

## Abstract

**Background:**

It is vital for residents to have a longitudinal view of their educational progression, and it is crucial for the medical education team to have a clear way to track resident progress over time. Current tools for aggregating resident data are difficult to use and do not provide a comprehensive way to evaluate and display resident educational advancement.

**Objective:**

This study aims to describe the creation and assessment of a system designed to improve the longitudinal presentation, quality, and synthesis of educational progress for trainees. We created a new system for residency progress management with 3 goals in mind, that are (1) a long-term and centralized location for residency education data, (2) a clear and intuitive interface that is easy to access for both the residents and faculty involved in medical education, and (3) automated data input, transformation, and analysis. We present evaluations regarding whether residents find the system useful, and whether faculty like the system and perceive that it helps them save time with administrative duties.

**Methods:**

The system was created using a suite of Google Workspace tools including Forms, Sheets, Gmail, and a collection of Apps Scripts triggered at various times and events. To assess whether the system had an effect on the residents, we surveyed and asked them to self-report on how often they accessed the system and interviewed them as to whether they found it useful. To understand what the faculty thought of the system, we conducted a 14-person focus group and asked the faculty to self-report their time spent preparing for residency progress meetings before and after the system debut.

**Results:**

The system went live in February 2022 as a quality improvement project, evolving through multiple iterations of feedback. The authors found that the system was accessed differently by different postgraduate years (PGY), with the most usage reported in the PGY1 class (weekly), and the least amount of usage in the PGY3 class (once or twice). However, all of the residents reported finding the system useful, specifically for aggregating all of their evaluations in the same place. Faculty members felt that the system enabled a more high-quality biannual clinical competency committee meeting and they reported a combined time savings of 8 hours in preparation for each clinical competency committee as a result of reviewing resident data through the system.

**Conclusions:**

Our study reports on the creation of an automated, instantaneous, and comprehensive resident progress management system. The system has been shown to be well-liked by both residents and faculty. Younger PGY classes reported more frequent system usage than older PGY classes. Faculty reported that it helped facilitate more meaningful discussion of training progression and reduced the administrative burden by 8 hours per biannual session.

## Introduction

Graduate medical education is governed by the Accreditation Council for Graduate Medical Education (ACGME). The ACGME criteria for compliance in all ACGME-accredited programs and successful graduation from one of these programs are required to be eligible to take certifying examinations. The requirements for these programs are vast [1] and cover content such as how resident evaluations should be conducted, the clinical responsibilities of residents, the make-up of the leadership team, the curriculum organization, and so on. In addition, the ACGME requires that educational files be created for current medical residents containing written evaluations from multiple evaluators, self-evaluations, mid- and end-year evaluations, rotation and training experience records, documentation of scholarly activity and quality improvement projects, and educational disciplinary actions and many other educational documents. The files can be maintained by paper or electronic records, but must be available for review easily and stored indefinitely as proof of training completion. These are mandated by the ACGME to ensure that proper training and documentation of training are available for all physicians. These files are difficult to create, maintain, organize, and ensure they remain easily accessible years later.

The current standard in residency education management is the New Innovations software platform [2]. However, the tool is not easy to use. For example, evaluations can be submitted on New Innovations only by users who have an account and are logged in. This can prohibit 360 evaluations from other individuals such as nursing staff and patients. There are many different types of reports, and both the formative and summative evaluations are shown individually instead of in a summarized manner, thereby inhibiting the ability to assess resident educational growth. There are limitations on what can be reported in real time, and no real dashboard to show missing information. There is no way to track board review and in-service training examination practice question completion. Some have tried to create shortcuts to enable easier data entry [3], but found no impact. Other departments are working toward a different platform for resident evaluation [4]. We know that the assessment of residents by faculty has a long-standing tradition [5], but finding a way to gather this information robustly and present it is a challenge. Building upon both the work for dashboard management and work around gathering high-quality faculty feedback by matching milestones with evaluation questions [6], we have developed a novel and fully automated system for residency progress management.

This system was designed to help residents and residency leadership have a clear view of the educational progress as well as milestones of the residents. In addition, it was designed to provide faculty involved in medical education a place to access all resident data for the ACGME-mandated clinical competency committee (CCC) meetings [7]. This study aims to introduce and describe the system, as well as provide evaluations regarding whether residents use and find it useful, and whether the faculty like the system and perceive that it helps them save time with administrative duties.

## Methods

### Resident Progress System

The resident progress system for all current Emergency Medicine trainees at Hackensack University Medical Center was initially completed and debuted in February 2022. After the system debuted, the study team gathered information from both Emergency Medicine residents and faculty involved in residency education regarding their views on the system.

The system was created through tight collaboration between the biomedical informatics and medical education division leadership within the Emergency Medicine department. The components of the system were decided upon through a needs-based discussion regarding which information was most pertinent to the residency (both the residents themselves and leadership) to holistically evaluate the rate of progress for individual residents, the ACGME accreditation requirements, and how this information should be best gathered. After a pilot phase where the system was enabled for residency administration, additional features were suggested and added. After the debut of the system for residents, more informal feedback was solicited and further additions were implemented. The system contains 14 of the 16 data types that CCC committees report using to assign milestone ratings to residents [8]. The 2 missing components are chart audits and simulated patient encounters (only used by <20% of residencies surveyed).

The system design work was focused on several goals in mind, that are (1) a long-term and centralized location for residency education data; (2) a clear and intuitive interface that is easy to access for both the residents and faculty involved in medical education; and (3) automated data input, transformation, and analysis.

**Figure 1:**
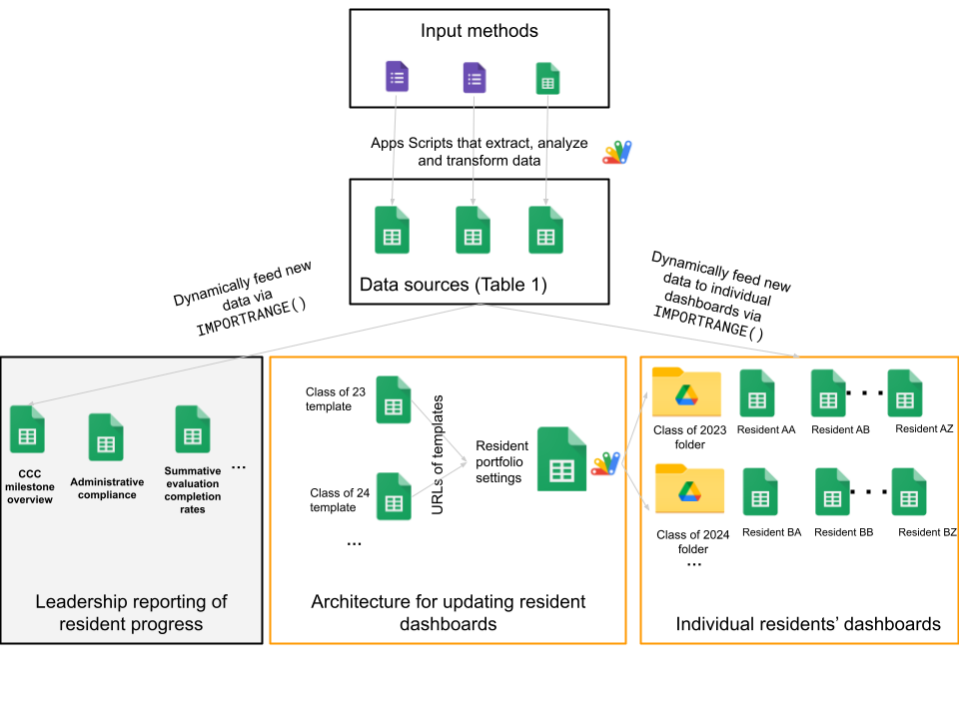
The technical architecture of the Resident progress system. The focus of the paper is on the individual residents’ dashboards, therefore the leadership reporting piece is greyed out. An example of the individual resident dashboards are presented in Figure 2 and the Multimedia Appendix 1. CCC: clinical competency committee.

**Figure 2:**
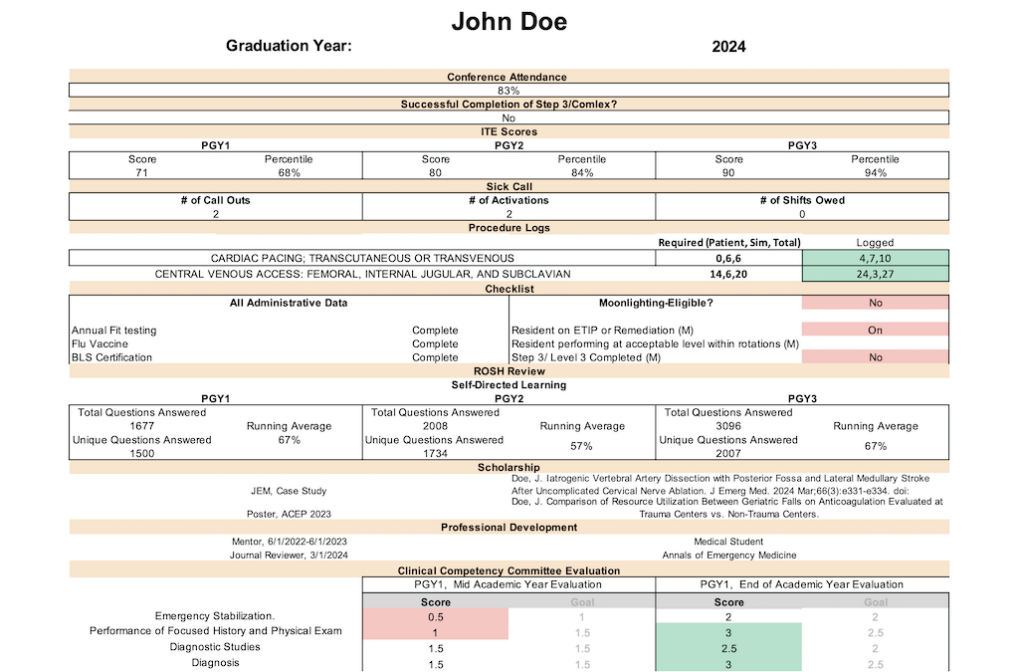
A shortened version of the two first tabs of an individual resident progress dashboard. A full example of a progress dashboard is presented in the Multimedia Appendix 1. PGY: postgraduate year.

The system involves sets of code that generate a leadership reporting view and individual resident progress dashboards based on a graduation-year template (Figure 1). Each resident’s progress dashboard takes the form of one Google Sheets workbook with a front page (Figure 2) and 17 standardized additional tabs for each resident. Within the dashboard, there is a total of 22 components represented across 3 categories (Table 1). A full example dashboard is provided in Multimedia Appendix 1. Google Forms are used to structure all evaluation responses and any other structured inputs, such as conference attendance, conference feedback, etc. Apps Script extensions are used for all programming aspects such as sending automated emails through Google.

**Table 1:** Standardized components of a resident dashboard.

Category and data		Data elements	Source (responsible party in italics)	Additional features		# of entries in a 2-year period^a^
**Administrative**						
	Sick calls	Call-outs, activations, shifts owed	A curated Google sheet for *chiefs* to mark scheduling changes		—^b^	Any time there is a sick call or call-out
	Dashboard attestations	Resident confirmation that portfolio was reviewed^c^	A Google form for *residents* to attest that they have reviewed the portfolio		—	69 attestations
	Administrative checklist	Status of compliance with administrative duties such as duty hours, Advanced Trauma Life Support certification, flu vaccine, step 3 successful completion, etc	A curated Google sheet for the *residency coordinator* to fill in		—	Any time there is an administrative change
	Conference attendance	Attendance at weekly residency conference	A curated Google sheet for *chiefs* to mark weekly attendance		—	Weekly attendance
	Scholarly work	Scholarship, service to the discipline, professional development, educational contributions^c^	A Google form with detailed instructions and branching logic to assist *residents* in developing CV-ready citations		Automatically compiles all scholarly activity for annual ACGME^d^ reporting	34 scholarship, 75 service, 24 professional, and 396 educational
**Assessment**						
	In-training examination score	Score and percentile	A curated Google sheet for the *residency coordinator* to fill in yearly, once scores are returned		—	Yearly, once in-training examination scores come out
	ROSH review	Self-directed learning, assigned questions	A transformed Google sheet that calculates individual statistics once *chiefs* import monthly ROSH data		—	—
	Procedure logs	Counts of required procedures^c^	A transformed Google sheet that automatically calculates individual statistics once *residency coordinator* imports monthly data from New Innovations		—	—
**Evaluations**						
	360 evaluations	Individual resident goals^c^	A Google form for working with *residents* on Specific, Measurable, Achievable, Relevant, Time-Bound goal setting		—	126 goal entries
	360 evaluations	Individual self-evaluations^c^	A Google form for walking *residents* through self-evaluations		—	119 self-evaluations
	360 evaluations	Clinical competency committee (CCC) evaluations	A transformed Google sheet filled out by *residency leadership* biannually during the CCC meeting to assign milestone achievement for each competency and each resident		All end-of-shift faculty feedback is aggregated automatically before each CCC meeting to provide data discussion	Biannual
	360 evaluations	Semiannual leadership evaluations	A Google form created for walking *residency leadership* through their semiannual meeting with each resident		If an evaluator submits a confidential comment, residency leadership is immediately sent an email to promote real-time follow-up	Biannual
	360 evaluations	End-of-shift faculty feedback	A Google Form for *attendings* to fill out after a clinical shift. Depending on the day of the week, the evaluator is about different clinical categories (task switching, diagnosis, etc). Each level (1-5) within each category is presented with an ACGME definition to ensure standardized evaluation		If an evaluator submits a confidential comment, residency leadership is immediately sent an email to promote real-time follow-up	2571 end-of-shift faculty evaluations
	360 evaluations	Peer feedback	A Google Form for generic assessment of peer *residents*		If an evaluator submits a confidential comment, residency leadership is immediately sent an email to promote real-time follow-up	180 peer evaluations
	360 evaluations	Patient evaluations	A Google Form for *patient* assessment of residents		If an evaluator submits a confidential comment, residency leadership is immediately sent an email to promote real-time follow-up	27 patient evaluations
	360 evaluations	Conference feedback	All *conference participants* are asked to evaluate each presentation, feedback data for each presenter is aggregated and presented in their portfolio		If an evaluator submits a confidential comment, residency leadership is immediately sent an email to promote real-time follow-up	4679 conference lecture evaluations
	360 evaluations	Pharmacist evaluations	A Google Form for *pharmacists* to evaluate residents		If an evaluator submits a confidential comment, residency leadership is immediately sent an email to promote real-time follow-up	3 pharmacist evaluations
	360 evaluations	Team member evaluations	A Google Form for all other *caregivers* to evaluate residents		If an evaluator submits a confidential comment, residency leadership is immediately sent an email to promote real-time follow-up	24 team member evaluations
	Summative evaluations	Individual for each Emergency Medicine rotation	A specialized Google form for Emergency medicine competency evaluation by *residency leadership*		If an evaluator submits a confidential comment, residency leadership is immediately sent an email to promote real-time follow-up	After each Emergency Medicine rotation
	Summative evaluations	Individual for each pediatric Emergency Medicine rotation	A specialized Google form for pediatric Emergency medicine competency evaluation by *pediatric EM leadership*		If an evaluator submits a confidential comment, residency leadership is immediately sent an email to promote real-time follow-up	After each Pediatric emergency medicine rotation
	Summative evaluations	Individual for each required outside rotation	A specialized Google form filled out by each *attending* contact person for each required outside rotation		If an evaluator submits a confidential comment, residency leadership is immediately sent an email to promote real-time follow-up	After each outside rotation
	Summative evaluations	Individual for each offered elective and selective	A specialized Google form for each offered elective and selective filled out by *attending* contact person for rotation		If an evaluator submits a confidential comment, residency leadership is immediately sent an email to promote real-time follow-up	After each selective rotation

^a^As different parts of the system went live at different times, we set it to a 24-month period since going live.

^b^Not applicable.

^c^Data that relies upon residents to self-report.

^d^ACGME: Accreditation Council for Graduate Medical Education.

Each resident is given view-only access to their own dashboard and residency leadership has view-only access to every resident’s dashboard. The biomedical informatics team members, who are responsible for programming the system, are the only ones with edit access.

As the entire system is created within the Google Workspace infrastructure and integrated with the organizational single sign-on authentication, the data are secure and protected. In addition, the entire system is fully reproducible for any department or institution using Google’s enterprise-wide secured platform.

The system evaluation was designed to understand the acceptance of the system in a quantitative and qualitative way among the faculty and residents. These evaluations were conducted as part of the system improvement process to understand the impact of the individual resident dashboards and gather information on how to improve them.

### Evaluation of the System by Residents

Residents were asked to self-report how often they look at their own dashboard. The residents were asked “How often do you access your residency dashboard?” with the multiple-choice answers of “Daily,” “Weekly,” “Monthly,” and “Once/Twice.” This data was collected by postgraduate year (PGY) year and recorded in a Google Sheet. The data were aggregated by PGY year and descriptive statistics were calculated. In addition, to gain a more complete picture of how the residents viewed the system, during their routine semiannual evaluations with residency leadership, they were asked to comment on how they liked the dashboard as a whole, what their favorite and least favorite parts were, and any changes they would suggest. The qualitative data on how much the residents liked the dashboards were aggregated and summarized.

### Evaluation of the System by Faculty

An unstructured focus group composed of all 14 CCC faculty members was used to gather qualitative information. Transcripts from the group were transcribed and assessed using thematic analysis. All faculty on the CCC were asked to self-report on time spent preparing for the CCC meeting before and after the debut of the resident system. These data were collected through a Google Form asking “Prior to or After implementation of the digital end-of-shift feedback form (using blue cards), on average how much time did you spend doing pre-work on all residents assigned to prepare for presentation in CCC meetings?” with the multiple-choice answers of “less than 1 hour,” “1-2 hours,” “2-4 hours,” and “more than 4 hours” and aggregated through Google Sheets to calculate pre-post count differences. The phrasing of the question was around the end-of-shift feedback because this was the first part of data collection that was moved to a digital platform and started the development of the entire residency progress system.

### Ethical Considerations

This work was conducted in the Hackensack University Medical Center Emergency Medicine Department. The Hackensack Meridian Health institutional review board approved this research study (Pro2022-0647). Waiver of informed consent was granted as the study involved a retrospective analysis of a quality improvement project (the creation of the Resident Progress system) and the data were gathered as part of the process of understanding whether the improvement project had the desired outcomes. For the data analysis purposes, all of the data were deidentified. All of the data were kept in a Google Workspace Drive (password-protected and encrypted by default), only accessible to key study personnel. There was no compensation offered to the participants.

## Results

### Resident Progress System

The progress system has been continuously live since February 2022. Through consistent iteration and feedback, new features have been added and the design has been altered. The robustness of the platform has been tested through the seamless integration of 2 new residency classes.

### Evaluation of the System by Residents

**Figure 3:**
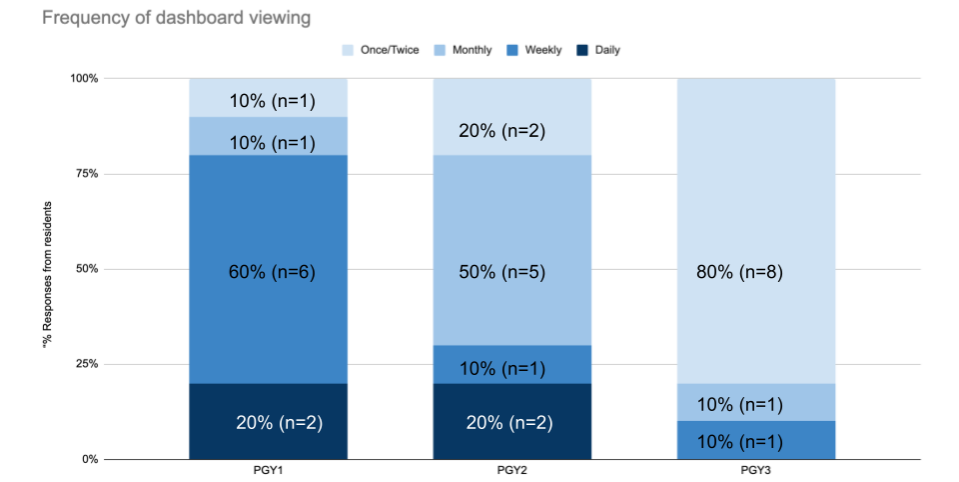
How residents answered the question “How often do you access your residency dashboard?” with multiple choice answers of: “Daily,” “Weekly,” “Monthly,” and “Once/Twice.” The response rates were as follows: 100% (10/10) of PGY1s, 90% (10/11) of PGY2s, and 83% (10/12) of PGY3s. PGY: postgraduate year.

Resident viewership and usage varied widely based on PGY level (Figure 3). PGY3s were less likely to make a habit of accessing the system, likely as they were already more than halfway through their final year when the system debuted and had their own workflows for accessing their data in other ways.

All residents reported finding the system useful. The most useful part was reported to be the aggregation of all data and more specifically, evaluations in 1 place (both formative-end of shift and summative-end of rotation and CCC milestone data). Some residents reported that the organization of the dashboard may be improved upon and potential inaccuracies when transitioning from 1 academic year to the next.

### Evaluation of the System by Faculty

Since the first version debuted, 5 CCC meetings were facilitated by the system. After the second CCC meeting, a focus group consisting of all CCC members was conducted. The focus group was centered around understanding the pros and cons of the previous process compared with the current one. Before the system, there was no unified place to find data on residents. In addition, there was no automated gathering of end-of-shift evaluations. The previous process for formative feedback consisted of residents handing faculty blue index cards (preprinted with questions regarding resident performance on shift) at the end of each shift. CCC members then collected the cards for review before the semiannual meeting.

The most robust theme that emerged was surrounding the quality of the previous process compared with the current one. This included subthemes of (1) formative feedback gathering at the end of the shift, (2) faculty preparation before the CCC meeting, and (3) organization of the day-long CCC meeting.

For the formative feedback gathering, the faculty felt that the new process solved the previous problem of poor handwriting (often made worse by the physical size constraint on the index cards). Of the members, one commented, “Reading other people’s handwriting was horrid.” Others agreed that in the previous method of handwritten feedback on blue index cards, legibility and adequate space for comments were consistent barriers to providing comprehensive feedback and reviewing it for CCC. One faculty member commented that they could “do more thorough feedback in a digital form because I can't scribble that much on the side [of the card]” and that the digital evaluation process “gives me a second to catch my breath and gather my thoughts”. There were some faculty who felt that the previous process resulted in more feedback: “I remember to fill out blue cards more.” and “The residents give us the blue cards more.” However, this may have been an outlier viewpoint as, over the course of a year, the digital feedback process has actually led to a 300% increase in the number of evaluations per resident, when compared with the blue index card method.

**Figure 4:**
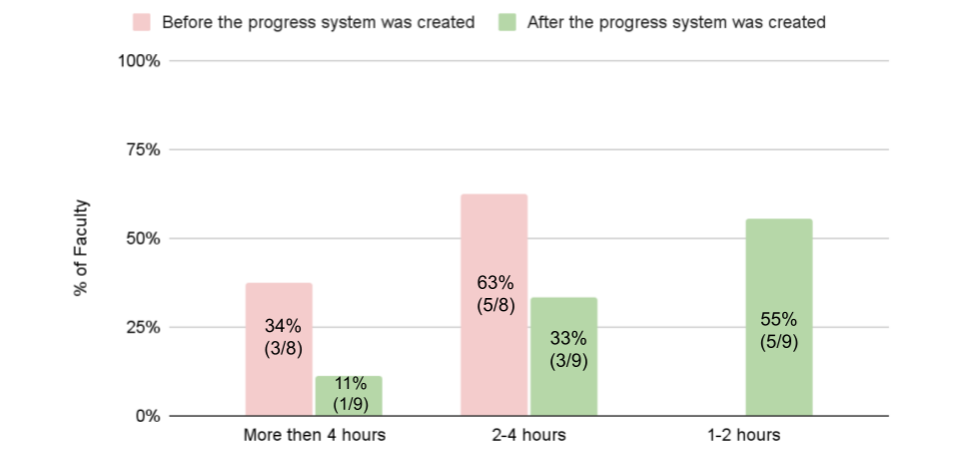
Self-reported time spent preparing for bi-annual CCC by each faculty member, before and after the system went live. This survey had a 64% (9/14) response rate. One participant did not answer the self-reported time spent before the intervention as this was their first CCC meeting. CCC: clinical competency committee.

For each CCC meeting, the CCC faculty members are assigned individual residents and asked to review their educational progress using a variety of data points (evaluations, test scores, procedure logs, etc). During the CCC meeting, each faculty member leads the discussion for their assigned residents. Regarding this preparation work, faculty said that the dashboard “... made preparing for the CCC a lot easier” and felt that they had more information to analyze about each resident. There was some discrepancy regarding the time spent for preparation, with some members saying they spent more time reviewing information: “...I feel like it took me longer to do. Just to flip through the tabs and go back and forth… I think I spent more time this time. Not because it's harder. It's just... There is more information”, while others reported huge time savings “It took half of the time”. In aggregate, there was a dramatic decrease in the amount of preparation time reported, with a total number of 8 hours saved (Figure 4).

With respect to the CCC meeting organization, the faculty felt that although the meeting took as much time as usual, the time was better spent: “...We spent the time on the people that needed to have it.” and “We spent a lot of time on people.” Notably, the third CCC meeting that was facilitated by the resident progress system actually finished an hour early, which was the first time that the CCC meeting finished ahead of schedule.

Finally, the focus group members were unanimous in their positive response to the system as a whole, with such comments about it as: “...The whole Google dashboard is awesome. That way you can see everything in one place,” “Yeah, it’s nice,” and “It’s amazing.”

## Discussion

### Principal Findings

Our study debuts and describes our novel resident progress system, consisting of both individual comprehensive resident portfolios and a set of leadership dashboards.

This study shows that our residency progress system has been a helpful tool for both residents and faculty alike. Both groups of users find the dashboard useful and report liking it. Faculty also reported a savings of 8 hours of preparation per biannual CCC meeting, due to the residency system’s aggregation and summarization of resident data in one place.

### Limitations

The authors recognize the limitation that not every institution and residency program will have an informatics division that can replicate the programming required to automate such a residency education management system. However, as the work does not use proprietary software but rather Google Workspace tools, it is a solution that is reproducible across other residency programs that have enterprise-wide versions of Google. In addition, we note that this system was created specifically for an Emergency Medicine department; however, the authors see no reason that such a system could not be applied and customized to other specialties and educational programs. Finally, much of the presented results are from self-reports. However, the authors also observed that the CCC meeting was run more fluidly and was completed more quickly, which can serve as another sign that the system is assisting with the biannual medical education discussions.

### Comparison With Previous Work

Although there are some examples of specialties creating their own system for learning management, these systems do not appear to still be in use [9]. In addition, there is a lot of work around portfolios and e-folios but they are geared more toward residents reflecting upon their education [10-13] through various points of their educational journey, whereas our study presents a more administratively focused residency management system. Our study is more comprehensive than anything automated currently reported in the literature. To date, there is no report in the graduate education literature that looks at the efficiency, ease of use, and accessibility of home-grown residency progress systems. We have programmed a fully automated system to capture, organize, summarize, and display individual residents’ relevant educational data. Our Resident Progress System not only uses a different mechanism for resident evaluation, but also collects and aggregates data across end-of-rotation evaluations, patient evaluations, peer evaluations, conference attendance, and in-training examination preparation and scores. We created the system as a way to reduce administrative burden and give residents transparency on expectations and their progress.

### Conclusions

The resident progress system has created a workflow that has shown to benefit both end users. Residents say the system is accessible and also report the uselfulness of the created system in its transparency in reporting their progress. The system has also shown to decrease the administrative burden on faculty in the form of hours saved compared with previous methods. We believe that this new way of presenting and synthesizing resident education tracking has been transformative for the department.

Residency leadership has also adopted the progress system for a variety of uses such as a tool to guide constructive conversation around strengths and areas for improvement during semiannual evaluations as well as a tool to streamline annual ACGME-mandated reporting requirements.

## Appendix

Multimedia Appendix 1 An example of an individual resident’s dashboard. The data presented is a combination of data from different resident’s dashboards and is edited and redacted to preserve privacy. To shorten the appendix, the following standard resident dashboard tabs have been removed: Pharmacist Evaluations, MICU PGY-1 Summative Evaluation, SICU Summative Evaluation, EM PGY-1 Summative Evaluation, EM PGY-2 Summative Evaluation, PEM PGY-1 Summative Evaluation, PEM PGY-2 Summative Evaluation, St. Croix Summative Evaluation, OB/GYN Summative Evaluation. Therefore, the frequency and volume are not representative of real resident dashboards.

## Abbreviations

ACGME: Accreditation Council for Graduate Medical Education
CCC: clinical competency committee
PGY: postgraduate year

## Data Availability

The transcripts of the focus group are not available due to concerns of reidentification. The aggregated data on self-reported usage are included in the published article.
