# Supplementary material for: Creation of an Automated and Comprehensive Resident Progress System for Residents and to Save Hours of Faculty Time: Mixed Methods Study
Source: JMIR Form Res. 2024 Sep 23;8:e53314. doi: 10.2196/53314 (PMC11459103; doi:10.2196/53314)
Supplement: Multimedia Appendix 1 [file formative_v8i1e53314_app1.pdf]

Most Recent Attestation of Portfolio Review
4/21/2023

John Doe

Graduation Year: 2024

Conference Attendance
83%

Successful Completion of Step 3/Comlex?
No

ITE Scores

PGY1		PGY2		PGY3	
Score	Percentile	Score	Percentile	Score	Percentile
71	68%	80	84%	90	94%

Sick Call

# of Call Outs	# of Activations	# of Shifts Owed
2	2	0

Procedure Logs

	Required (Patient, Sim, Total)	Logged
CARDIAC PACING; TRANSCUTANEOUS OR TRANSVENOUS	**0,6,6**	4,7,10
CENTRAL VENOUS ACCESS: FEMORAL, INTERNAL JUGULAR, AND SUBCLAVIAN	**14,6,20**	24,3,27
CRICOTHYROTOMY	**0,3,3**	0,7,7
DISLOCATION REDUCTION OF FRACTURE	**10,0,10**	21,0,21
INTUBATIONS	**24,11,35**	49,10,59
LUMBAR PUNCTURE	**10,5,15**	17,5,22
PERICARDIOCENTESIS	**0,3,3**	0,2,2
PROCEDURAL SEDATION	**10,5,15**	16,0,16
RESUSCITATION: ADULT MEDICAL & NONTRAUMATIC SURGICAL	**30,15,45**	110,7,117
RESUSCITATION: ADULT TRAUMA	**24,11,35**	147,2,149
RESUSCITATION: PEDIATRIC MEDICAL	**10,5,15**	10,5,15
RESUSCITATION: PEDIATRIC TRAUMA	**7,3,10**	20,2,22
THORACOSTOMY TUBE INSERTION	**7,3,10**	11,5,15
VAGINAL DELIVERY OF NEWBORN	**10,0,10**	9,1,10

Checklist

All Administrative Data		Moonlighting-Eligible?	No
Annual Fit testing	Complete	Resident on ETIP or Remediation (M)	No
Flu Vaccine	Complete	Resident performing at acceptable level within rotations (M)	Yes
BLS Certification	Complete	Step 3/ Level 3 Completed (M)	Yes
ACLS Certification	Complete	Performing at acceptable level in all aspects - Milestones (M)	No
PAL Certification	Needs Renewal	ITE Percentage 50% or higher (M)	Yes
ATLS Certification	Complete	Rosh Review in compliance (M)	Complete
Live Required training course	Complete	Signed up or completed annual conference lectures (M)	Complete
Learning Modules	Complete	Conference Attendance 70% or higher (M)	Yes
Duty Hours Q1	Complete	Duty Hour up to date (M)	Complete
Duty Hours Q2	Complete	Procedure Log up to date (M)	Complete
Duty Hours Q3	Complete	Scholarly activity deadlines have been met (M)	Incomplete
Duty Hours Q4	N/A	Learning Modules up to date (M)	Complete
NJ Permit Application	Complete	Peer, self, and end of rotation evaluations (M)	Complete
New Innovations - Reappointment packet	Complete	Academic Affairs Moonlighting application (M)	Incomplete
New Innovations - Onboarding packet	Complete		

Notes: Review and sign your reappointment contract in New innovations , complete scholarly activity

Last Updated: 7/19/2023

ROSH Review

Self-Directed Learning

	PGY1		PGY2		PGY3	
Total Questions Answered	1677		2008		3096	
Running Average		67%		57%		67%
Unique Questions Answered	1500		1734		2007	

Assigned Questions

Running Average

PGY1	56%
PGY2	66%
PGY3	65%

Monthly Scores

July	2022	71%
August	2022	70%
Sept	2022	63%
Oct	2022	70%
Nov	2022	62%
Dec	2022	54%

	Jan	2023
	Feb	2023
	Mar	2023
	Apr	2023
	May	2023
	Jun	2023
	Jul	2023

Last Updated: 7/19/2023

Scholarship	
JEM, Case Study	Doe, J. Iatrogenic Vertebral Artery Dissection with Posterior Fossa and Lateral Medullary Stroke After Uncomplicated Cervical Nerve Ablation. J Emerg Med. 2024 Mar;66(3):e331-e334. doi: 10.1016/j.
Poster, ACEP 2023	Doe, J. Comparison of Resource Utilization Between Geriatric Falls on Anticoagulation Evaluated at Trauma Centers vs. Non-Trauma Centers.

Service	
Committee, Hospital Wide, Mortalities	member
Committee, Department Wide, 72 Hour Returns	member

Professional Development	
Mentor, 6/1/2022-6/1/2023	Medical Student
Journal Reviewer, 3/1/2024	Annals of Emergency Medicine

Educational	
Lecture, 07/1/2021	Lateral MI
Lecture, 04/13/2022	Nasal Fracture Reduction
Lecture, 01/25/2023	Minor Eye Emergencies
InSitu Sim, 03/29/2022	Paracentesis
Morning Report, 12/09/2022	Intussusception
Lecture, 11/09/2022	Morbidity and Mortality Conference
Morning Report, 08/9/2022	Mouth Ulcers
InSitu Sim, 08/04/2023	Covert Snakebit
InSitu Sim, 02/07/2024	Pediatric SVT

John Doe

Clinical Competency Committee Evaluations

	PGY1, Mid Academic Year Evaluation		PGY1, End of Academic Year Evaluation		PGY2, Mid Academic Year Evaluation		PGY2, End of Academic Year Evaluation		PGY3, Mid Academic Year Evaluation		PGY3, End of Academic Year Evaluation	
	**Score**	Goal	**Score**	Goal	**Score**	Goal	**Score**	Goal	**Score**	Goal	**Score**	Goal
Emergency Stabilization.	0.5	1	2	2	3	2.5	4	3	4	3.5	4.5	4.5
Performance of Focused History and Physical Exam	1	1.5	3	2.5	3	3	4	3.5	4.5	4	4.5	5
Diagnostic Studies	1.5	1.5	2.5	2	3	3	3.5	3.5	4	4	4.5	4.5
Diagnosis	1.5	1.5	3	2.5	3	3	4	3.5	4	4	4.5	4.5
Pharmacotherapy	1.5	1.5	2	2	2.5	2.5	3	3	4	3.5	5	4
Reassessment and Disposition	1.5	1.5	2.5	2.5	3.5	3	4.5	4	4.5	4.5	5	5
Task Switching	2	2	2.5	2.5	3.5	3	4	3.5	4.5	4.5	5	5
General Approach to Procedures	1	2	2	2.5	3	3	3	3.5	4.5	4.5	5	5
Scientific Knowledge	1	1	2	2.5	3.5	3.5	3.5	4	4.5	4.5	5	5
Treatment & Clinical Reasoning	1	1.5	3	2.5	4	3	4	3.5	4	4	5	4.5
Patient Safety	1	2	2.5	2.5	3	3	3.5	3.5	4.5	4.5	5	5
Quality Improvement	1.5	1.5	2.5	2.5	3	3	3.5	3.5	4.5	4.5	5	5
System Navigation for Patient-Centered Care	1	1.5	2.5	2	3	3	3.5	3.5	4	4	4	4.5
Physician Role in Healthcare Systems	0.5	1	2	2	3	3	3.5	3.5	4	4	4	4.5
Evidence-Based and Informed Practice	1	1	1.5	1.5	2.5	2.5	3.5	3.5	4.5	4.5	5	5
Reflective Practice and Commitment to Personal Growth	1.5	2	2.5	3	3.5	3.5	4	4	4.5	4.5	5	5
Professional Behavior and Ethical Principles	1	1	2	2	3	3	4	3.5	4.5	4.5	5	5
Accountability / Conscientiousness	1.5	1.5	2.5	2	3	2.5	3	3	4.5	4	5	5
Self-awareness and Well-being	2	1.5	2.5	2.5	3	3	3.5	3.5	4	4	5	5
Patient and Family Centered Communication	1	1	2	2	3.5	3	4	3.5	4.5	4.5	4.5	5
Interprofessional and Team Communication	1	1	2	2	3.5	3	3.5	3.5	4	4	5	4.5
Communication within healthcare systems	2	1.5	2.5	2.5	3.5	3	4	3.5	4	4	4.5	4.5

Semi-Annual Evaluations

	PGY1, Mid Academic Year Evaluation	PGY1, End of Academic Year Evaluation	PGY2, Mid Academic Year Evaluation	PGY2, End of Academic Year Evaluation	PGY3, Mid Academic Year Evaluation	PGY3, End of Academic Year Evaluation
**Growth Plan**	Good start to the residency		John is making thoughtful goals. We talked at length about this.	John's wellness goal is to complete more of documentation on shift	John has set very realistic and achievable goals for his last 6 months as a resident.	John has blossomed this year. PGY3 about to graduate!
**Milestone Evals**						
**Patient Care**	Exceeds Expectations	Exceeds Expectations	Exceeds Expectations	Exceeds Expectations	Exceeds Expectations	Exceeds Expectations
**Practice Based Learning**	Meets Expectations	Exceeds Expectations	Meets Expectations	Meets Expectations	Meets Expectations	Meets Expectations
**Medical Knowledge**	Meets Expectations	Meets Expectations	Meets Expectations	Meets Expectations	Meets Expectations	Meets Expectations
**Professionalism**	Meets Expectations	Exceeds Expectations	Exceeds Expectations	Meets Expectations	Meets Expectations	Exceeds Expectations
**Interpersonal and Communication Skills**	Meets Expectations	Exceeds Expectations	Meets Expectations	Meets Expectations	Meets Expectations	Meets Expectations
**System-Based Practice**	Meets Expectations	Meets Expectations	Meets Expectations	Meets Expectations	Meets Expectations	Exceeds Expectations
**Comments on milestone areas of improvement**			John is at and above level. Functions independently	na		
**Non-ACGME Items**						
**Rosh Review Questions**	Meets Expectations	Meets Expectations	Meets Expectations	Exceeds Expectations	Meets Expectations	Exceeds Expectations
**ITE Score**	Exceeds Expectations	Exceeds Expectations	Needs Improvement	Needs Improvement	Meets Expectations	Meets Expectations
**CITI Modules**	Meets Expectations	Meets Expectations	Meets Expectations	Meets Expectations		
**Duty Hours**	Meets Expectations	Needs Improvement	Meets Expectations	Meets Expectations		
**Administrative Compliance**	Exceeds Expectations	Meets Expectations	Meets Expectations	Meets Expectations	Meets Expectations	Meets Expectations
**Scholarly Activity and/or QI**	Meets Expectations	Meets Expectations	Meets Expectations	Meets Expectations	Meets Expectations	Meets Expectations
**Comments on non-ACGME areas of improvement**			ITE last year was a little bit on the lower side.	John is going to focus on different methods of studying for ITE this year to bring up percentile.		
**Other Comments**						
**Procedure Comments**			On track	Numbers in common procedures are fine.	Logging procedures appropriately	All set to graduate.
**General Summary and Action Plan**						
**Decision Regarding training progression**	Continue as expected	Continue as expected	Continue to train at current level	Continue training at current PGY year (for mid year) or progress to next training year (end of year)	Continue as expected	Continue training at current PGY year (for mid year) or progress to next training year (end of year)
**Is the resident at risk of any of the following?**	None	None	No	No	None	None
**Comments (goals or risks for next evaluation period)**		Keep it up! You are doing a great job.	Overall Action items for the next 6 months: finish notes on shift.	John has excelled	John received a lot of positive praise in CCC.	Congrats on everything you've accomplished over the last 3 years!

John Doe

Individual Goals

	PGY1, Mid Academic Year Evaluation	PGY1, End of Academic Year Evaluation	PGY2, Mid Academic Year Evaluation	PGY2, End of Academic Year Evaluation	PGY3, Mid Academic Year Evaluation	PGY3, End of Academic Year Evaluation
**Wellness Goal**						
Please list your Wellness Goal	[redacted]		[redacted]	[redacted]	[redacted]	[redacted]
Please explain how this goal is specific.	[redacted]		[redacted]	[redacted]		[redacted]
Please explain how you will measurable this goal.	[redacted]		[redacted]			[redacted]
Please explain how you will attain this goal.	[redacted]		[redacted]	[redacted]	[redacted]	
Please explain how this is relevant to the type of physician you want to be when you are done with residency.	[redacted]		[redacted]	[redacted]	[redacted]	[redacted]
What is the time frame you want to achieve this by? This will serve as a time your mentor will try to keep you accountable.	[redacted]			[redacted]	[redacted]	[redacted]
**Medical Knowledge Goal**						
Please list your Medical Knowledge Goal	[redacted]		[redacted]	[redacted]	[redacted]	[redacted]
Please explain how this goal is specific.	[redacted]		[redacted]	[redacted]	[redacted]	[redacted]
Please explain how you will measurable this goal.			[redacted]	[redacted]	[redacted]	[redacted]
Please explain how you will attain this goal.	[redacted]		[redacted]	[redacted]	[redacted]	[redacted]
Please explain how this is relevant to the type of physician you want to be when you are done with residency.	[redacted]		[redacted]	[redacted]	[redacted]	[redacted]
What is the time frame you want to achieve this by? This will serve as a time your mentor will try to keep you accountable.	[redacted]		[redacted]	[redacted]	[redacted]	[redacted]
**Clinical Practice Goal**						
Please list your Clinical Practice Goal	[redacted]		[redacted]		[redacted]	[redacted]
Please explain how this goal is specific.	[redacted]		[redacted]		[redacted]	[redacted]
Please explain how you will measurable this goal.	[redacted]		[redacted]		[redacted]	[redacted]
Please explain how you will attain this goal.	[redacted]		[redacted]		[redacted]	[redacted]
Please explain how this is relevant to the type of physician you want to be when you are done with residency.	[redacted]		[redacted]		[redacted]	[redacted]
What is the time frame you want to achieve this by? This will serve as a time your mentor will try to keep you accountable.	[redacted]		[redacted]		[redacted]	[redacted]
**Professional Development Goal**						
Please list your Professional Development Goal	[redacted]		[redacted]	[redacted]		[redacted]
Please explain how this goal is specific.			[redacted]	[redacted]		[redacted]
Please explain how you will measurable this goal.	[redacted]		[redacted]	[redacted]		[redacted]
Please explain how you will attain this goal.	[redacted]		[redacted]	[redacted]		[redacted]
Please explain how this is relevant to the type of physician you want to be when you are done with residency.	[redacted]		[redacted]	[redacted]		[redacted]
What is the time frame you want to achieve this by? This will serve as a time your mentor will try to keep you accountable.	[redacted]		[redacted]	[redacted]		[redacted]

John Doe

Self-Evaluations

	PGY1, Mid Academic Year Evaluation	PGY1, End of Academic Year Evaluation	PGY2, Mid Academic Year Evaluation	PGY2, End of Academic Year Evaluation	PGY3, Mid Academic Year Evaluation	PGY3, End of Academic Year Evaluation
**Patient Care**			**Score**			
I can obtain a reliable history and physical.	4	4	4	4	5	5
I consider resource utilization and cost when choosing tests for patients.	3	3	3	3	4	4
I provide a safe and complete signout at the end of shift.	4	4	4	4	4	5
I have an adequate fund of knowledge for my training level.	4	4	4	4	4	5
I know when to ask for help with patient care.	5	5	5	5	5	5
I am comfortable recognizing and stabilizing critically ill patients.	4	4	4	4	4	5
**Systems-based Practice**			**Score**			
I am a thoughtful team player who works well with others.	5	5	4	5	5	5
I prioritize important tasks and I am efficient.	4	4	4	4	4	5
I am an effective team leader.	4	4	3	4	4	4
**Interpersonal and Communication Skills**			**Score**			
I communicate effectively with patients, attendings, consultants, nurses and other team members.	4	4	4	4	4	5
I am an effective teacher.	3	3	3	3	4	4
**Professionalism**			**Score**			
I demonstrate compassion, integrity, respect for patients and staff, and accept responsibility for my actions and work.	5	5	5	5	5	5
I consistently complete assigned administrative and educational tasks.	5	5	4	5	5	5
I remain respectful and cooperative during group activities and express myself in a professional manner.	5	5	5	5	5	5
**Burnout and Job Satisfaction**			**Score**			
I feel emotionally drained from my work.	2	3	3	4	3	2
I feel fatigued when I get up in the morning.	2	3	3	4	3	2
Working with people is really a strain.	2	3	3	3	3	2
I have become more callous and cynical since starting residency.	2	3	2	3	3	2
I do not care what happens to some patients.	1	1	1	1	1	1
I treat some patients as impersonal objects.	2	2	1	2	2	2
I feel exhilarated working with patients.	3	3	3	3	3	3
I have accomplished many things.	4	4	3	3	4	4
I have positively influenced others' lives.	4	4	4	4	4	4

Category	Comments
**Patient Care**	[redacted]
**System-Based Practice**	
**Interpersonal and Communication Skills**	
**Professionalism**	
**Something that really makes me feel burned out is**	
**General**	

John Doe

Faculty Feedback

Date	Evaluator	CCC Time Frame	Comment
6/21/2023	[redacted]	2023 Mid Academic Year Evaluation	[redacted]
6/21/2023	[redacted]	2023 Mid Academic Year Evaluation	[redacted]
6/15/2023	[redacted]	2023 Mid Academic Year Evaluation	[redacted]
6/15/2023	[redacted]	2023 Mid Academic Year Evaluation	[redacted]
6/9/2023	[redacted]	2023 Mid Academic Year Evaluation	[redacted]
6/2/2023	[redacted]	2023 Mid Academic Year Evaluation	[redacted]
6/2/2023	[redacted]	2023 Mid Academic Year Evaluation	[redacted]
5/31/2023	[redacted]	2023 Mid Academic Year Evaluation	[redacted]
3/28/2023	[redacted]	2023 End of Academic Year Evaluation	[redacted]
3/15/2023	[redacted]	2023 End of Academic Year Evaluation	[redacted]
3/14/2023	[redacted]	2023 End of Academic Year Evaluation	[redacted]
3/9/2023	[redacted]	2023 End of Academic Year Evaluation	[redacted]
3/6/2023	[redacted]	2023 End of Academic Year Evaluation	[redacted]
3/4/2023	[redacted]	2023 End of Academic Year Evaluation	[redacted]
2/18/2023	[redacted]	2023 End of Academic Year Evaluation	[redacted]
2/14/2023	[redacted]	2023 End of Academic Year Evaluation	[redacted]
2/3/2023	[redacted]	2023 End of Academic Year Evaluation	[redacted]
2/3/2023	[redacted]	2023 End of Academic Year Evaluation	[redacted]
2/2/2023	[redacted]	2023 End of Academic Year Evaluation	[redacted]
2/2/2023	[redacted]	2023 End of Academic Year Evaluation	[redacted]
2/1/2023	[redacted]	2023 End of Academic Year Evaluation	[redacted]
1/30/2023	[redacted]	2023 End of Academic Year Evaluation	[redacted]
1/27/2023	[redacted]	2023 End of Academic Year Evaluation	[redacted]
1/27/2023	[redacted]	2023 End of Academic Year Evaluation	[redacted]
1/22/2023	[redacted]	2023 End of Academic Year Evaluation	[redacted]
1/12/2023	[redacted]	2023 End of Academic Year Evaluation	[redacted]
1/8/2023	[redacted]	2023 End of Academic Year Evaluation	[redacted]
1/6/2023	[redacted]	2023 End of Academic Year Evaluation	[redacted]
12/26/2022	[redacted]	2023 End of Academic Year Evaluation	[redacted]
12/25/2022	[redacted]	2023 End of Academic Year Evaluation	[redacted]
12/24/2022	[redacted]	2023 End of Academic Year Evaluation	[redacted]
12/18/2022	[redacted]	2023 End of Academic Year Evaluation	[redacted]
		2022 Mid Academic Year Evaluation	[redacted]
		2022 Mid Academic Year Evaluation	[redacted]
		2022 Mid Academic Year Evaluation	[redacted]

John Doe

Patient Evaluations

	PGY1, Mid Academic Year Evaluation	PGY1, End of Academic Year Evaluation	PGY2, Mid Academic Year Evaluation	PGY2, End of Academic Year Evaluation	PGY3, Mid Academic Year Evaluation	PGY3, End of Academic Year Evaluation
**# of Patient-Evaluations FIlled out about me**	**3**	**1**	**2**	**3**	**2**	**2**
**Patient Safety**	**% Yes**	**% Yes**	**% Yes**	**% Yes**	**% Yes**	**% Yes**
Did your physician introduce themselves fully?	100%	100%	100%	100%	100%	100%
Did your physician confirm your name and date of birth with your ID band?	33%	100%	100%	100%	100%	100%
**Patient Care**	**Median**	**Median**	**Median**	**Median**	**Median**	**Median**
Did your physician make you feel comfortable?	5	4	5	5	5	4
Did you physician answer all your questions?	5	5	4	5	5	5
How well do you feel your physician listened to your concerns?	4	4	5	4	5	5
How well do you feel your physician explained the treatment plan to you?	3	4	4	4	5	5
**Comments**						
General Comments						Listened to my concerns and did not interupt me

John Doe

Peer Evaluations

	PGY1, Mid Academic Year Evaluation			PGY1, End of Academic Year Evaluation			PGY2, Mid Academic Year Evaluation			PGY2, End of Academic Year Evaluation			PGY3, Mid Academic Year Evaluation			PGY3, End of Academic Year Evaluation		
**# of Peer-Evaluations FIlled out**											**6**							
**# of Peer-Evaluations FIlled out about me**								**1**			**2**						**2**	
**Patient Care**	**Median**	**Max**	**Min**	**Median**	**Max**	**Min**	**Median**	**Max**	**Min**	**Median**	**Max**	**Min**	**Median**	**Max**	**Min**	**Median**	**Max**	**Min**
I can rely on the history and physical obtained by this resident.							5	5	5	4.5	5	4				5	5	5
This resident knows when to ask for help.							5	5	5	4.5	5	4				5	5	5
This resident provides a safe signout at the end of shift.							5	5	5	4	4	4				5	5	5
**System-Based Practice**	**Median**	**Max**	**Min**	**Median**	**Max**	**Min**	**Median**	**Max**	**Min**	**Median**	**Max**	**Min**	**Median**	**Max**	**Min**	**Median**	**Max**	**Min**
This resident is a team player who works well with others.							5	5	5	5	5	5				5	5	5
This resident prioritizes important tasks and is efficient.							5	5	5	4	4	4				5	5	5
**Interpersonal and Communication Skills**	**Median**	**Max**	**Min**	**Median**	**Max**	**Min**	**Median**	**Max**	**Min**	**Median**	**Max**	**Min**	**Median**	**Max**	**Min**	**Median**	**Max**	**Min**
This resident communicates effectively with patients, attendings, consultants, nurses and other team members.							4	4	4	4	4	4				5	5	5
This resident is an effective teacher.							5	5	5	3.5	4	3				4.5	5	4
**Professionalism**	**Median**	**Max**	**Min**	**Median**	**Max**	**Min**	**Median**	**Max**	**Min**	**Median**	**Max**	**Min**	**Median**	**Max**	**Min**	**Median**	**Max**	**Min**
This resident demonstrates compassion, integrity, respect for patients and staff, and accepts responsibility for their actions and work.							5	5	5	5	5	5				5	5	5
**Comments**																		
General Comments							John continues to improve every shift.			John is a great, dedicated physician. Good team player------						---Extremely thorough and hard-working-John is very efficient and able to handle a high workload.		

John Doe

Conference Lecture Feedback

lonth of Conference Lectur	Lecture Title	verage Scor	Comments
			Conference Year 2021-2022
03/09/2022	Facial Fractures	4.0	- Enjoyed the presentation! Would recommend talking more loudly.<br>- Mostly a review of anatomy. Not always clinically-relevant. Delivery could be improved<br>-more eye contact, more engagement<br>-I liked the questions at the end to consolidate everything that you taught.
			Conference Year 2022-2023
1/18/2023	Brugada Syndrome	4	- No comments
12/7/2022	Hyperthyroidism	4.7	Great presentation, professionally put together, up to date info and kept my attention--good<br>-Great talk<br>-excellent refresher, I like how you described hyperthyroidism as a spectrum<br>-Was able to use some of these teaching points with patients
	Animal and Vector Bourne Illness	4.4	-clear and comprehensive. i gotta remember hantavirus!<br>-n/a<br>-<br>-succinct but detailed<br>-Great thorough review before ITE
11/23/2022	EKGs: STEMI	4.1	- Informative<br>- nice job<br>- Good review and reinforcement of posterior STEMI<br>- good thorough with explanation of types of MI<br>- excellent lecture<br>- N/A<br>- great review<br>- Good job!
10/19/2022	CCB Toxicity	4.4	- Comprehensive and went into good details about the backstory, but would have preferred more ECGs
			Conference Year 2023-2024
5/22/2024	se of PPI in Acute Upper GI Ble	5	- Good job<br>- Nicely organized, explained easily, chock full of useful info.<br>- Good job! Great review of important research points and posing the clinical question of utility in the end. Consider doing a case presentation
4/10/2024	Femur, Knee Fractures	5	- Good pics

John Doe

Team Member Evaluations

	PGY1, Mid Academic Year Evaluation			PGY1, End of Academic Year Evaluation			PGY2, Mid Academic Year Evaluation			PGY2, End of Academic Year Evaluation			PGY3, Mid Academic Year Evaluation			PGY3, End of Academic Year Evaluation		
**# of Team Member-Evaluations FIlled out about me**	**1**			**1**						**1**			**2**					
**Patient Care**	**Median**	**Max**	**Min**	**Median**	**Max**	**Min**	**Median**	**Max**	**Min**	**Median**	**Max**	**Min**	**Median**	**Max**	**Min**	**Median**	**Max**	**Min**
The resident listens to patients effectively and allows patients to ask questions.				5	5	5				5	5	5	5	5	5			
The resident educates patients regarding conditions, tests, management and treatment in words patient can understand.				5	5	5				5	5	5	5	5	5			
The resident is attentive to patient comfort.				5	5	5				5	5	5	5	5	5			
If procedures were performed, were they done safely? Does the resident update nurses, patients, and families on the status (changes/delays) of the are plan?				5	5	5				5	5	5	5	5	5			
**System-Based Practice**	**Median**	**Max**	**Min**	**Median**	**Max**	**Min**	**Median**	**Max**	**Min**	**Median**	**Max**	**Min**	**Median**	**Max**	**Min**	**Median**	**Max**	**Min**
The resident advocates for the patient in the healthcare system.				4	4	4					0	0	5	5	5			
The resident calls PMD, family members, case management or other resources when needed.				5	5	5				5	5	5	5	5	5			
**Interpersonal and Communication Skills**	**Median**	**Max**	**Min**	**Median**	**Max**	**Min**	**Median**	**Max**	**Min**	**Median**	**Max**	**Min**	**Median**	**Max**	**Min**	**Median**	**Max**	**Min**
The resident communicates clearly.	3	3	3	5	5	5				5	5	5	5	5	5			
The resident is willing to answer questions and provide explanations.	3	3	3	5	5	5				5	5	5	5	5	5			
The resident negotiates and compromises when disagreements occur.	3	3	3	5	5	5					0	0	5	5	5			
**Professionalism**	**Median**	**Max**	**Min**	**Median**	**Max**	**Min**	**Median**	**Max**	**Min**	**Median**	**Max**	**Min**	**Median**	**Max**	**Min**	**Median**	**Max**	**Min**
The resident demonstrate compassion and honesty to patients and their families.				4	4	4				5	5	5	5	5	5			
The resident demonstrate respect, and integrity to staff, nurses, and colleagues.				5	5	5				5	5	5	5	5	5			
The resident responds promptly when paged or called.				5	5	5				5	5	5	5	5	5			
**Comments**																		
General Comments	Struggling with proper communication with staff especially to provide rationale for treatments.			His approach is with not only patients but his staff members are very positive. This doctor is personable, knowledgable and has great bedside manner.						Dr Doe has always communicated clear patient care plans and is attentive to patients' concerns. Tkes time to lsiten.			He has respect and such a great attitude. It is always a pleasure to work with him.					

John Doe

MICU PGY-3 Summative Evaluation

Milestone	3/6/2023		
**Patient Care**			
Demonstrate competence in performing an adequate history and appropriate exam for the critically ill adult patient.	Exceeds expectations		
Evaluate and provide appropriate ongoing management of patients in the critical care setting, and supervise transitions of care into and out of the ICU.	Exceeds expectations		
Perform as a team leader during assessment of patients who are decompensating and new admissions during arrival.	Meets expectations		
Demonstrate competence with point of care ultrasound in the daily evaluation of the critical patient..	Exceeds expectations		
Demonstrate competence in the direction of cardiac arrest resuscitation, including following ACLS guidelines, assigning roles, and evaluating the patient for etiology of arrest.	Meets expectations		
Demonstrate understanding of ventilator settings, recommend adjustments to settings based on changes in patient physiology or vital signs, manage complications of patients on ventilators, and troubleshoot common ventilator alarms.	Meets expectations		
Demonstrate ability to diagnose and treat shock, sepsis, fluid and electrolyte abnormalities, cardiac failure, cardiac dysrhythmias, renal failure, hepatic failure, and toxicologic emergencies.	Exceeds expectations		
Demonstrate appropriate prioritization of diagnostic and therapeutic interventions in critical patients.	Meets expectations		
Interpret and change management based on data from ECG monitors, ECGs, cardiac outputs, hemodynamic monitoring, arterial blood gases, pulse oximetry, end tidal CO2 monitors and ventilators..	Meets expectations		
Demonstrate the ability to work in a multi-disciplinary team to form treatment plans for critically ill patients in a systematic way.	Exceeds expectations		
Understand the indication and appropriateness of de-escalation of care and the role of end of life care discussions with critically ill patients and family members.	Exceeds expectations		
Did you answer "Does not meet expectations" to any of the above questions?	No		
For any domains that the resident did not meet expectations, please provide an explanation.			
**Medical Knowledge**			
Discuss the pathophysiology of illnesses that require critical care, and teach junior team members at an appropriate level.	Exceeds expectations		
Understand, implement and educate the team on current protocols for treatment of critical patients.	Meets expectations		
Manage fluid and electrolyte disturbances.	Meets expectations		
State the indications/contraindications for use of invasive cardiac monitoring and central venous access.	Meets expectations		
Describe the dosages, indications and contraindications of pharmacologic interventions for shock, cardiac failure, dysrhythmias, sepsis, trauma, toxins, respiratory failure, hepatic failure, renal failure, and neurologic illnesses.	Meets expectations		
Did you answer "Does not meet expectations" to any of the above questions?	No		
For any domains that the resident did not meet expectations, please provide an explanation.			
**Practice-Based Learning and Improvement**			
Identify critical treatments that must be given in a timely fashion prior to the patient being transferred to the intensive care unit.	Meets expectations		
Appraise and assimilate evidence from scientific studies related to patients' health problems, and facilitate learning of junior residents.	Meets expectations		
Identify areas for improvement in fund of knowledge or patient care abilities and initiate improvement activities, as needed.	Exceeds expectations		
Develop treatment plans in conjunction with other consultants, non-physician team members, patients and their families.	Meets expectations		
Did you answer "Does not meet expectations" to any of the above questions?	No		
For any domains that the resident did not meet expectations, please provide an explanation.			
**Interpersonal and Communication Skills**			
Demonstrate ability to compassionately and appropriately communicate with patients and their family members.	Exceeds expectations		
Effectively communicate with patients' nurses and other team members regarding changes in condition and planned interventions.	Exceeds expectations		
Develop the skills of effectively giving bad news such as poor prognosis or death to family members of critical patients and discussion of DNR status.	Exceeds expectations		
With appropriate guidance, direct family meetings with identified surrogates while acting as a patient advocate.	Meets expectations		
Did you answer "Does not meet expectations" to any of the above questions?	No		
For any domains that the resident did not meet expectations, please provide an explanation.			
**Professionalism**			
Develop and maintain interpersonal, and communication skills essential to interactions with patients, family, and staff.	Exceeds expectations		
Practice ethical decision making with cultural sensitivity.	Meets expectations		
Did you answer "Does not meet expectations" to any of the above questions?	No		
For any domains that the resident did not meet expectations, please provide an explanation.			
**System-Based Learning**			
Understand the importance and roles of multiple caregivers in a typical academic ICU setting.	Meets expectations		
Demonstrate an understanding of the ethical and legal principles applicable to the care of critically ill patients.	Meets expectations		
Demonstrate understanding of "do not resuscitate" orders, advance directives, living wills and brain death criteria.	Meets expectations		
Understand quality measures related to cost and patient care within the critical care unit.	Meets expectations		
Did you answer "Does not meet expectations" to any of the above questions?	No		

For any domains that the resident did not meet expectations, please provide an explanation.			
Please comment on the resident's overall performance.	It was great working with John! He manages ICU patient appropriately, asks questions when needed and guides his team. Exceeded my expectations. He really did a phenomenal job!		

John Doe

Trauma Summative Evaluation

Milestone	6/12/2023	12/14/2021
**Patient Care**		
Demonstrate competence in performing an adequate general and appropriately focused trauma exam.	Meets expectations	Meets expectations
Demonstrate ability to perform an adequate history and physical examination related to the chief complaint as observed or confirmed by an attending physician or senior resident.	Exceeds expectations	Meets expectations
Accurately present each patient to an attending physician or senior resident and offer several diagnostic possibilities for each clinical problem seen.	Exceeds expectations	Meets expectations
Recognize presentations or circumstances threatening to life or limb and to immediately report such patients to an attending physician or senior resident.	Exceeds expectations	Meets expectations
Document an appropriate assessment and management plan for each chief complaint presented by a patient.	Exceeds expectations	Meets expectations
Demonstrate competence in performing an adequate and appropriate neurologic exam on trauma patients with various levels of consciousness.	Exceeds expectations	Meets expectations
Demonstrate the ability to recognize and evaluate respiratory and airway emergencies in trauma patients.	Meets expectations	Meets expectations
Understand the complexities of care planning for elderly or chronically ill patients that present with traumatic injuries.	Exceeds expectations	Meets expectations
Did you answer "Does not meet expectations" to any of the above questions?	No	No
For any domains that the resident did not meet expectations, please provide an explanation.		
**Medical Knowledge**		
Understand the principles of trauma management and ATLS guidelines.	Exceeds expectations	Meets expectations
Formulate a differential diagnosis based on clinical findings for altered mental status including chemical, psychological, and organic causes.	Meets expectations	Meets expectations
Demonstrate an understanding of the evaluation and management of hemorrhagic hypovolemic shock.	Exceeds expectations	Meets expectations
Describe the indications and utility of various modalities to evaluate complaints of shortness of breath including the diagnoses of pneumothorax, hemothorax, flail chest, and pulmonary embolism.	Exceeds expectations	Meets expectations
Describe the indications and utility of various modalities to evaluate complaints of chest pain including transection of the aorta, pneumothorax, pulmonary contusion, cardiac contusion, cardiac tamponade, and rib fractures.	Exceeds expectations	Meets expectations
Correctly request and interpret radiographic studies for complaints of extremity pain and trauma.	Exceeds expectations	Meets expectations
Correctly request CT studies for evaluation of traumatic presentations.	Exceeds expectations	Meets expectations
Understand the indications for trauma surgery consultations and indications for operative management.	Meets expectations	Meets expectations
Did you answer "Does not meet expectations" to any of the above questions?	No	No
For any domains that the resident did not meet expectations, please provide an explanation.		
**Practice-Based Learning and Improvement**		
Develop skills in the work-up of the patient with a medical problem based upon evidence-based medicine and apply these skills in the ordering of appropriate laboratory, radiological, and ancillary studies.	Meets expectations	Meets expectations
Apply knowledge of study designs and statistical methods to the appraisal of clinical studies and other information on diagnostic and therapeutic effectiveness.	Meets expectations	Meets expectations
Identify areas for improvement in their fund of knowledge or patient care abilities.	Meets expectations	Meets expectations
Facilitate learning of current EM principles and practices from colleagues, health care professionals, and with the use of references, on-line materials, journal articles and discussion.	Exceeds expectations	Meets expectations
Did you answer "Does not meet expectations" to any of the above questions?	No	No
For any domains that the resident did not meet expectations, please provide an explanation.		
**Interpersonal and Communication Skills**		
Succinctly and efficiently request and communicate with consultants for trauma patients requiring specialty management.	Meets expectations	Meets expectations
Demonstrate appropriate and complete documentation of patients' encounters.	Meets expectations	Meets expectations
Develop and maintain interpersonal, and communication skills essential to interactions with patients, family, and staff.	Meets expectations	Meets expectations
Did you answer "Does not meet expectations" to any of the above questions?	No	No
For any domains that the resident did not meet expectations, please provide an explanation.		
**Professionalism**		
Develop and maintain teamwork skills essential to interactions with staff, patients and family.	Exceeds expectations	Meets expectations
Practice ethical decision making with cultural sensitivity.	Meets expectations	Meets expectations
Practice medicine in a fashion that displays competence, consideration, and integrity.	Exceeds expectations	Meets expectations
Function as a team member in medical and trauma resuscitations.	Exceeds expectations	Meets expectations
Did you answer "Does not meet expectations" to any of the above questions?	No	No

For any domains that the resident did not meet expectations, please provide an explanation.		
**System-Based Learning**		
Demonstrate the importance of follow-up care and continuity of care for the trauma patient and the role of the EM physician to facilitate that within the healthcare system.	Meets expectations	Meets expectations
Appropriately access healthcare and social needs for patients.	Meets expectations	Meets expectations
Provide cost effective management and patient care including cost appropriate medications and treatment modalities.	Meets expectations	Meets expectations
Demonstrate knowledge of cost containment, resource allocation, quality of care and access to care issues as it pertains to trauma patients and elderly trauma patients.	Meets expectations	Meets expectations
Discuss rules and regulations relating to EMTALA, HIPAA, and patient's rights as it pertains to trauma patients.	Meets expectations	Meets expectations
Did you answer "Does not meet expectations" to any of the above questions?	No	No
For any domains that the resident did not meet expectations, please provide an explanation.		
Please comment on the resident's overall performance.	Reliable member of the team. Excellent presentations with plans.	excellent

John Doe

EM PGY-3 Summative Evaluation

Milestone	8/9/2023	3/28/2023
**Patient Care**		
Independently evaluate, treat, and plan final disposition on non critical Emergency Department patients. All cases must be discussed with and evaluated by an EM attending physician prior to final disposition.	Meets expectations	Meets expectations
Monitor and improve overall patient flow, address intra-departmental or extra-departmental issues which impede patient care and flow, and supervise EM1 residents, off-service residents, and medical students.	Meets expectations	Meets expectations
Perform as a team leader in all resuscitations. Designate specific personnel to perform critical procedures during resuscitations.	Meets expectations	Meets expectations
Perform initial EMS patient evaluations to help determine initial severity of illness, direct patient placement within the Emergency Department based on perceived severity of illness, and decide on activation of the trauma, stroke, or cardiac catheterization teams in conjunction with supervision from the attending physician.	Meets expectations	Meets expectations
Arrange for evaluation by consultants prior to case discussion with the EM attending and base subsequent actions on their recommendations and other input.	Meets expectations	Meets expectations
Demonstrate the ability to manage multiple patients simultaneously, including high acuity patients.	Meets expectations	Meets expectations
Demonstrate EM3 equivalent competence performing a focused history and physical examination including proper use of the history and exam to minimize need for unnecessary testing, recognizing uncommon presentations in complex patients and escalating care appropriately.	Meets expectations	Meets expectations
Demonstrate appropriate treatment priorities, identifying patients by acuity.	Meets expectations	Meets expectations
Competently perform and teach emergency medicine procedures with minimal supervision and provide feedback to learners for such procedures.	Meets expectations	Meets expectations
Independently recognize and manage uncommon and complex complications of common procedures.	Meets expectations	Meets expectations
Did you answer "Does not meet expectations" to any of the above questions?	No	No
For any domains that the resident did not meet expectations, please provide an explanation.		
**Medical Knowledge**		
Demonstrate the ability to teach junior learners how to seek out clinical guidelines and journal articles that are pertinent to their patients' presentations.	Meets expectations	Meets expectations
Demonstrate the ability to teach and educate junior residents and medical students.	Meets expectations	Meets expectations
Design and implement senior research and/or quality projects with faculty mentorship.	Meets expectations	Meets expectations
Understand the pathophysiology and principles of treatment of uncommon emergent and non emergent disease states that present to the emergency department.	Meets expectations	Meets expectations
Understand the indications and contraindications to uncommon emergent procedural interventions that are required in the emergency department and how to address uncommon complications of these procedures.	Meets expectations	Meets expectations
Demonstrate thorough knowledge of resuscitation protocols and understand the process of change and implementation of these protocols.	Meets expectations	Meets expectations
Demonstrate correct interpretation of all utilized radiographic studies in emergency medicine.	Meets expectations	Meets expectations
Demonstrate knowledge of a variety of medications used to treat uncommon emergent and non-emergent patient presentations in the emergency department including the mechanism of action, onset of action and potential side effects.	Meets expectations	Meets expectations
Did you answer "Does not meet expectations" to any of the above questions?	No	No
For any domains that the resident did not meet expectations, please provide an explanation.		
**Practice-Based Learning and Improvement**		
Prepare and present morning reports during the academic year with supervisional guidance from assigned faculty.	Meets expectations	Meets expectations
Prepare and present three (3) core lectures during the academic year at Emergency Medicine Conference with supervisional guidance from faculty that lead each curriculum.	Meets expectations	Meets expectations
Present a Morbidity and Mortality Grand Rounds style presentation during weekly Emergency Medicine Conference once during the academic year.	Meets expectations	Meets expectations
Facilitate learning of current EM principles and practices with colleagues, healthcare professionals, and through the use of references, on-line materials, journal articles and discussion.	Meets expectations	Meets expectations
Identify areas for improvement in fund of knowledge or patient care abilities and initiate improvement activities, as needed.	Meets expectations	Meets expectations
Did you answer "Does not meet expectations" to any of the above questions?	No	No
For any domains that the resident did not meet expectations, please provide an explanation.		
**Interpersonal and Communication Skills**		
Demonstrate superior communication skills with patients, visitors, other residents, attendings and other hospital personnel.	Meets expectations	Meets expectations
Develop clinical teaching skills when supervising junior level residents.	Meets expectations	Meets expectations
Succinctly and efficiently request appropriate consultation for patients requiring specialty management.	Meets expectations	Meets expectations
Maintain accurate, comprehensive and timely medical documentation of patient encounters.	Meets expectations	Meets expectations
Educate and counsel patients and families on treatment plans, potential diagnoses, self care, return precautions, and follow up plans.	Meets expectations	Meets expectations
Did you answer "Does not meet expectations" to any of the above questions?	No	No

For any domains that the resident did not meet expectations, please provide an explanation.		
**Professionalism**		
Develop the ability to recognize and intervene with colleagues and peers when problems are identified that may put patients at risk.	Meets expectations	Meets expectations
Develop and maintain teamwork skills essential to interactions with staff, patients and family.	Meets expectations	Meets expectations
Timely completion of all patient encounter notes. Specifically all notes completed within 24 hours from the end of any given shift.	Meets expectations	Meets expectations
Demonstrate appropriate chart documentation and medical decision making that reflects the level of an EM3 with the goal of attending level documentation of medical decision making.	Meets expectations	Meets expectations
Did you answer "Does not meet expectations" to any of the above questions?	No	No
For any domains that the resident did not meet expectations, please provide an explanation.		
**System-Based Learning**		
See approximately 2 patients per hour depending on complexity and acuity of the patient.	Meets expectations	Meets expectations
Demonstrate the ability to manage multiple patients simultaneously, including high acuity cases.	Meets expectations	Meets expectations
Demonstrate an ability to manage EM flow by assisting with triage, prioritizing and appropriately expediting patient care as needed.	Meets expectations	Meets expectations
Manage administrative problems as they arise in conjunction with the supervising attending.	Meets expectations	Meets expectations
Supervise junior house staff (EM and off service residents) and medical students. Junior house staff and medical students may present patients to the EM3, who directs and manages patient care and conducts bedside teaching.	Meets expectations	Meets expectations
Understand the indications, appropriate utilization and need specific instances for involvement of consultants within the context of Emergency Medicine practice and assist in the teaching of those indications to junior staff while on shift.	Meets expectations	Meets expectations
Determine safe and effective disposition plans including appropriate levels of inpatient care, indications for transfer to higher levels of care, as well as organization of outpatient care and follow up plans.	Meets expectations	Meets expectations
Did you answer "Does not meet expectations" to any of the above questions?	No	No
For any domains that the resident did not meet expectations, please provide an explanation.		
Please comment on the resident's overall performance.	Functions at level, has great composure on shift	He is doing well and functioning at or above level as a PGY3 and meeting all expectations.

John Doe

Pediatric EM PGY-3 Summative Evaluation

Milestone	3/29/2023	
**Patient Care**		
Manage pediatric medical and surgical patients that present to the Emergency Department under the supervision of an emergency department attending.	Exceeds expectations	
Begin directing pediatric trauma resuscitations.	Exceeds expectations	
Demonstrate ability to acquire a history and physical exam, develop a broad differential of pediatric disorders and formulate a treatment plan appropriate to the EM level of training.	Exceeds expectations	
Discuss the diagnostic work-up and disposition when child abuse and/or neglect are suspected.	Exceeds expectations	
Demonstrate management of patients with upper airway infection, pediatric exanthem, altered mental status, cardiac arrest, airway obstruction, ALTE, burns, lower airway disease, diabetes, neonatal jaundice, bleeding disorder, GI complaints, urologic complaints, and other common conditions.	Exceeds expectations	
Did you answer "Does not meet expectations" to any of the above questions?	No	
For any domains that the resident did not meet expectations, please provide an explanation.		
**Medical Knowledge**		
Demonstrate knowledge of the significance of fever in children of various ages.	Exceeds expectations	
Demonstrate knowledge of common infectious diseases of childhood, including appropriate work-up and treatment of meningitis, sepsis, pneumonia, urinary tract infection, and bacteremia.	Exceeds expectations	
Calculate fluid and electrolyte requirements of a dehydrated child and maintenance with IV fluids.	Exceeds expectations	
Demonstrate knowledge of the pathophysiology and manifestations of common and/or serious disease of the GI tract, upper and lower respiratory tract, limp, testicular and penile disorders, poisonings, ingestions.	Exceeds expectations	
Discuss the differences in the management and differential of newborns, toddlers, children and adolescents and how it differs for adults.	Exceeds expectations	
Know the correct algorithm for PALS and ATLS and the correct resuscitation medications.	Exceeds expectations	
Did you answer "Does not meet expectations" to any of the above questions?	No	
For any domains that the resident did not meet expectations, please provide an explanation.		
**Practice-Based Learning and Improvement**		
Locate, appraise, and utilize scientific evidence for the patient's health problems and the larger population from which they are drawn.	Exceeds expectations	
Facilitate learning of current Pediatric EM principles and practices from colleagues, health care professionals, and with the use of references, on-line materials, journal articles and discussions.	Exceeds expectations	
Develops skills in the work-up of the patient with a medical problem based upon evidence-based medicine and apply these skills in the ordering of appropriate laboratory, radiological, and ancillary studies.	Exceeds expectations	
Did you answer "Does not meet expectations" to any of the above questions?	No	
For any domains that the resident did not meet expectations, please provide an explanation.		
**Interpersonal and Communication Skills**		
Demonstrate appropriate and complete documentation of patients' encounters.	Exceeds expectations	
Develop and maintain interpersonal, and communication skills essential to interactions with patients, family, and staff.	Exceeds expectations	
Understand that communicating with the parents and family as well as the child are integral components to the pediatric patients welfare.	Exceeds expectations	
Learn how to communicate with pediatric patients of various ages.	Exceeds expectations	
Demonstrate ability to interact with violent Emergency Department pediatric patients and discuss protection techniques for other patients, families and staff members.	Exceeds expectations	
Did you answer "Does not meet expectations" to any of the above questions?	No	
For any domains that the resident did not meet expectations, please provide an explanation.		
**Professionalism**		
Practice ethical decision making with cultural sensitivity in children and understand how cultural values can impact care decisions.	Exceeds expectations	
Care for the pediatric patient in a fashion that displays competence, consideration, and integrity.	Exceeds expectations	
Develop and maintain teamwork skills essential to interactions with staff, patients and family.	Exceeds expectations	
Timely completion of all patient encounter notes. Specifically all notes completed within 24 hours from the end of any given shift.	Exceeds expectations	
Did you answer "Does not meet expectations" to any of the above questions?	No	
For any domains that the resident did not meet expectations, please provide an explanation.		
**System-Based Learning**		
Interact with pediatricians, primary care physicians, patients and families in coordinating the care of the pediatric patient.	Exceeds expectations	
Understand how public health care policies and reporting requirements affect care in the Pediatric Emergency Department.	Exceeds expectations	

Know the hospital system for dealing with violent patients, patients with special needs, patients with psychiatric disease or family members.	Exceeds expectations	
Understand the role of social worker, child welfare workers, legal regulations and proper disposition when child abuse and/or neglect is suspected.	Exceeds expectations	
Did you answer "Does not meet expectations" to any of the above questions?	No	
For any domains that the resident did not meet expectations, please provide an explanation.		
Please comment on the resident's overall performance.	John has continued to grow throughout his three years	

John Doe

Community EM Summative Eval

Milestone	12/10/2021		
**Patient Care**			
Understand patient flow and treatment under the supervision of an attending in a community Emergency Department setting	Meets expectations		
Demonstrate the ability to rapidly evaluate multiple patients, prioritize their care and manage their treatments.	Meets expectations		
Demonstrate the ability to evaluate and manage patients of all ages that commonly present to a community Emergency Department.	Meets expectations		
Demonstrate the ability to manage the acutely ill patient and conduct the required resuscitation.	Meets expectations		
Appropriately refer patients for follow-up care and assist patients in access to continuity of care.	Meets expectations		
Did you answer "Does not meet expectations" to any of the above questions?	No		
For any domains that the resident did not meet expectations, please provide an explanation.			
**Medical Knowledge**			
Discuss a broad differential for abdominal pain, chest pain, shortness of breath, weakness and other common complaints in the Emergency Department.	Meets expectations		
Understand the sensitivity and specificity of ancillary studies in the Emergency Department.	Meets expectations		
Describe the clinical presentation, etiologies, pathophysiology and current therapy for stroke, acute coronary syndrome, acute congestive heart failure, sepsis, seizure, pneumonia, and intracranial hemorrhage.	Meets expectations		
Did you answer "Does not meet expectations" to any of the above questions?	No		
For any domains that the resident did not meet expectations, please provide an explanation.			
**Practice-Based Learning and Improvement**			
Locate, appraise, and utilize scientific evidence to affect patient's health problems and the larger population from which they are drawn.	Meets expectations		
Facilitate learning of current EM principles and practices from colleagues, health care professionals, use of references, on-line materials, journal articles and discussion.	Meets expectations		
Identify areas for improvement with regards to fund of knowledge or patient care abilities.	Meets expectations		
Did you answer "Does not meet expectations" to any of the above questions?	No		
For any domains that the resident did not meet expectations, please provide an explanation.			
**Interpersonal and Communication Skills**			
Develop and maintain strong interpersonal, and communication skills essential to interactions with patients, family, and staff.	Exceeds expectations		
Demonstrate ability to effectively and appropriately communicate with consultants, other health care professionals and staff.	Meets expectations		
Did you answer "Does not meet expectations" to any of the above questions?	No		
For any domains that the resident did not meet expectations, please provide an explanation.			
**Professionalism**			
Practice ethical decision making with cultural sensitivity for the patients of this community hospital.	Meets expectations		
Understand and apply principles of professionalism, ethics, and legal concepts pertinent to patient management.	Meets expectations		
Did you answer "Does not meet expectations" to any of the above questions?	No		
For any domains that the resident did not meet expectations, please provide an explanation.			
**System-Based Learning**			
Interact with the various private physicians in the community and understand safe inter-institutional transfer.	Meets expectations		
Demonstrate knowledge of cost containment, resource allocation, quality of care and access to care issues.	Meets expectations		
Did you answer "Does not meet expectations" to any of the above questions?	No		
For any domains that the resident did not meet expectations, please provide an explanation.			
Please comment on the resident's overall performance.	Dr. Doe was a hard working resident		

EMS Summative Eval

Milestone	3/17/2022		
**Patient Care**			
Understand the impact and barriers to delivery of care in the pre-hospital and their effects on patient care within the healthcare system and the Emergency Department.	Meets expectations		
Assist patients to access acute and immediate health care.	Meets expectations		
Demonstrate competence in caring for the patient in the pre-hospital setting with paramedics and other pre-hospital healthcare providers during a ride-along in the ambulance.	Meets expectations		
Demonstrate competence in applying the principles of triage and emergency medical care delivery in the pre-hospital setting.	Meets expectations		
Demonstrate skills in spinal and extremity immobilization as indicated.	Meets expectations		
Did you answer "Does not meet expectations" to any of the above questions?	No		
For any domains that the resident did not meet expectations, please provide an explanation.			

Medical Knowledge			
Know the ACLS, asthma, COPD, CHF medications and interventions given in pre-hospital care.	Meets expectations		
Know and review ACLS and ATLS principles and protocols.	Meets expectations		
Demonstrate accurate EKG and rhythm strip reading skills.	Meets expectations		
Describe common environmental, toxicological, and biological hazards encountered in the pre-hospital care setting as well as injury prevention techniques.	Meets expectations		
Did you answer "Does not meet expectations" to any of the above questions?	No		
For any domains that the resident did not meet expectations, please provide an explanation.			
**Practice-Based Learning and Improvement**			
Prepare and present one educational activity to paramedic students and other learners within the HackensackUMC EMS system.	Meets expectations		
Review one EMS case for quality review.	Meets expectations		
Locate, appraise, and utilize scientific evidence to patient's health problems and the larger population from which they are drawn with an observation of pre-hospital care.	Meets expectations		
Did you answer "Does not meet expectations" to any of the above questions?	No		
For any domains that the resident did not meet expectations, please provide an explanation.			
**Interpersonal and Communication Skills**			
Succinctly and efficiently present the patient's pre-hospital presentation and course to the hospital healthcare provider.	Meets expectations		
Demonstrate effective listening skills and be able to elicit and provide information using verbal, nonverbal, and written skills.	Meets expectations		
Provide appropriate patient education in the area of accessing the healthcare system.	Meets expectations		
Demonstrate the teamwork required to interact with a pre-hospital EMS provider.	Meets expectations		
Did you answer "Does not meet expectations" to any of the above questions?	No		
For any domains that the resident did not meet expectations, please provide an explanation.			
**Professionalism**			
Develop and maintain interpersonal, and communication skills essential to interactions with patients, family, and staff during the pre-hospital care.	Meets expectations		
Practice medicine in a fashion that displays competence, consideration, and integrity.	Meets expectations		
Protect patient's and family member's confidentiality during the pre-hospital care.	Meets expectations		
Did you answer "Does not meet expectations" to any of the above questions?	No		
For any domains that the resident did not meet expectations, please provide an explanation.			
**System-Based Learning**			
Understand the environment of the pre-hospital EMS system.	Meets expectations		
Understand the medical, regulatory, administrative and organizational aspects of pre-hospital care.	Meets expectations		
Discuss the educational requirements and skill levels of different levels of EMS provider and licensure.	Meets expectations		
Understand the importance of continuity in the pre-hospital to hospital transition of care in the Emergency Department patient.	Meets expectations		
Discuss medico-legal liability issues relating to EMS.	Meets expectations		
Demonstrate understanding of the common organizational structures of Emergency Medical Services Systems.	Meets expectations		
Demonstrate understanding of appropriate utilization practices for ground and air medical services.	Meets expectations		
Discuss the importance of, and the various methods for, medical control in EMS systems.	Meets expectations		
Define the national components of EMS disaster management.	Meets expectations		
Demonstrate an understanding of the unique aspects and limitations of the pre-hospital environment.	Meets expectations		
Did you answer "Does not meet expectations" to any of the above questions?	No		
For any domains that the resident did not meet expectations, please provide an explanation.			
Please comment on the resident's overall performance.	Resident completed the 4 required shifts		

PICU Summative Eval

Milestone	12/15/2021		
**Patient Care**			
Perform the initial evaluation and stabilization of the critically ill pediatric patient.	Meets expectations		
Rapidly identify patients with acute life-threatening illnesses including traumatic injuries and medical emergencies.	Meets expectations		
Learn to interpret radiographs and laboratory results, and understand their relationship to a patient's clinical presentation.	Meets expectations		
Acquire, if possible, a final diagnosis for a pediatric patient, based on the integration of the patient's (and maternal) history, review of systems, physical examination, laboratory and radiographic data, and other diagnostic test results.	Meets expectations		
Understand and manage the unique respiratory needs of the pediatric patient and develop an understanding of the principles guiding the use of mechanical ventilators and CPAP.	Meets expectations		
Understand and manage the unique nutritional requirements of the critically ill pediatric patient.	Meets expectations		
Learn indications, contraindications, and complications of, and develop proficiency with airway management, central line placement, and intra-arterial catheterization in the pediatric patient.	Meets expectations		
Did you answer "Does not meet expectations" to any of the above questions?	No		

For any domains that the resident did not meet expectations, please provide an explanation.			
**Medical Knowledge**			
Describe the etiology, presentation, pathophysiology, and treatment of the diseases encountered during this rotation, as appropriate for the resident's level of training.	Meets expectations		
Develop an understanding of the natural history of diseases of the critically ill pediatric patient.	Meets expectations		
Did you answer "Does not meet expectations" to any of the above questions?	No		
For any domains that the resident did not meet expectations, please provide an explanation.			
**Practice-Based Learning and Improvement**			
Understand the educational resources available in the hospital, including the textbooks, computer-based programs and internet resources applicable to pediatric critical care.	Meets expectations		
Demonstrate the ability to form a clinical question and use the available resources to resolve patient care issues.	Meets expectations		
Did you answer "Does not meet expectations" to any of the above questions?	No		
For any domains that the resident did not meet expectations, please provide an explanation.			
**Interpersonal and Communication Skills**			
Develop effective presentation and communication skills in order to transmit succinct, accurate clinical information to other health care practitioners.	Meets expectations		
Develop effective and professional methods of interaction with other health care providers on the pediatric service and consultants.	Meets expectations		
Develop a professional relationship with patients and their families in order to maximize physician-patient information exchange, the family's involvement in the treatment plan, and trust in the plan of care.	Meets expectations		
Develop a working relationship with residents, medical students, nurses, support staff, and attending pediatric intensivists in the PICU.	Meets expectations		
Did you answer "Does not meet expectations" to any of the above questions?	No		
For any domains that the resident did not meet expectations, please provide an explanation.			
**Professionalism**			
Learn to clearly and accurately document the patient record.	Meets expectations		
Provide patient care that incorporates sensitivity to the patient's critical status, ethnic, and social background and understand how these factors may influence the plan of care.	Meets expectations		
Understand the role of the physician as an advocate for the patient in the current medical environment.	Meets expectations		
Did you answer "Does not meet expectations" to any of the above questions?	No		
**System-Based Learning**			
Function as an integral member of the PICU team.	Meets expectations		
Learn and understand the indications for PICU admission and discharge to a less acute care setting.	Meets expectations		
Understand how medical, social, legal, ethical, and economic considerations may influence the provision and delivery of health care.	Meets expectations		
Understand the role of the pediatric intensivist in the evaluation of patients in the ED.	Meets expectations		
Did you answer "Does not meet expectations" to any of the above questions?	No		
For any domains that the resident did not meet expectations, please provide an explanation.			
Please comment on the resident's overall performance.	Was sometimes less present in the afternoon and seemed less interested than other residents		

US PGY-3 Summative Eval

Milestone	5/30/2023		
**Patient Care**			
Demonstrate and teach proper technique and indications for point of care ultrasound in the practice of emergency medicine.	Exceeds expectations		
Demonstrate competency in the routine point of care emergency ultrasounds (POCUS) such as FAST, AAA, cardiac, biliary, renal, DVT, first trimester pregnancy, venous access, procedural, soft tissue, ocular, advanced cardiac and testicular ultrasound.	Exceeds expectations		
Demonstrate ability to recognize and differentiate adequate from inadequate studies of recorded images for continuous quality improvement, and assist intern and medical students who are on rotation.	Exceeds expectations		
Demonstrate ability to correctly interpret EUS findings and share them with the team caring for the patient.	Exceeds expectations		
Did you answer "Does not meet expectations" to any of the above questions?	No		
For any domains that the resident did not meet expectations, please provide an explanation.			
**Medical Knowledge**			
Understand the principles of ultrasound physics and so they may be used to enhance image acquisition.	Exceeds expectations		
Understand both indications and limitations of EUS.	Exceeds expectations		
Know the landmarks required for adequate views of each exam.	Meets expectations		
Describe and recognize normal vs abnormal images.	Exceeds expectations		
List the pathologies that can be recognized on POCUS and the sensitivity and specificity of the exam for each.	Exceeds expectations		
Recognize the different types of ultrasound artifacts and apply this knowledge to inadequate views by junior team members.	Exceeds expectations		

Did you answer "Does not meet expectations" to any of the above questions?	No		
For any domains that the resident did not meet expectations, please provide an explanation.			
**Practice-Based Learning and Improvement**			
Effectively use the educational resources available in the ED, including on-line texts and databases to enhance understanding of EUS.	Exceeds expectations		
Identify areas for self-improvement and implement strategies to enhance sonographic knowledge, skills, and processes of care.	Exceeds expectations		
Did you answer "Does not meet expectations" to any of the above questions?	No		
For any domains that the resident did not meet expectations, please provide an explanation.			
**Interpersonal and Communication Skills**			
Develop and consistently demonstrate listening skills to address patients' concerns respectfully and effectively.	Exceeds expectations		
Ensure that patients (and their families) understand the nature of the studies being performed.	Exceeds expectations		
Develop effective methods for communicating with colleagues in the emergency department regarding scans performed on scanning shifts.	Exceeds expectations		
Did you answer "Does not meet expectations" to any of the above questions?	No		
For any domains that the resident did not meet expectations, please provide an explanation.			
**Professionalism**			
Demonstrate empathy and compassion toward patients and their families.	Exceeds expectations		
Emulate the role of the emergency physician as a patient advocate.	Exceeds expectations		
Incorporate sensitivity for the patient's age, gender, sexual orientation, religious beliefs, and socioeconomic background in providing them care in the ED.	Exceeds expectations		
Respect patient autonomy, comfort and modesty as you perform sonographic studies.	Exceeds expectations		
Appropriately document results of studies and link images to orders so they are preserved in the electronic medical record.	Exceeds expectations		
Did you answer "Does not meet expectations" to any of the above questions?	No		
For any domains that the resident did not meet expectations, please provide an explanation.			
**System-Based Learning**			
Demonstrate appropriate use of ED resources.	Exceeds expectations		
Integrate sonographic findings into the care of patients appropriately.	Exceeds expectations		
Did you answer "Does not meet expectations" to any of the above questions?	No		
For any domains that the resident did not meet expectations, please provide an explanation.			
Please comment on the resident's overall performance.	John is a rock star.		
